# Facial hair may slow detection of happy facial expressions in the face in the crowd paradigm

**DOI:** 10.1038/s41598-022-09397-1

**Published:** 2022-04-08

**Authors:** Barnaby J. W. Dixson, Tamara Spiers, Paul A. Miller, Morgan J. Sidari, Nicole L. Nelson, Belinda M. Craig

**Affiliations:** 1 School of Psychology, The University of Queensland, St. Lucia, QLD 4067 Australia; 2 School of Health and Behavioural Sciences, University of the Sunshine Coast, Sippy Downs, 4502 Australia; 3 School of Psychology, University of Adelaide, Adelaide, SA 5005 Australia; 4 School of Population Health, Curtin University, Bentley, WA 6102 Australia; 5 Faculty of Health Sciences and Medicine, Bond University, Robina, QLD 4229 Australia

**Keywords:** Psychology, Human behaviour

## Abstract

Human visual systems have evolved to extract ecologically relevant information from complex scenery. In some cases, the face in the crowd visual search task demonstrates an anger superiority effect, where anger is allocated preferential attention. Across three studies (*N* = 419), we tested whether facial hair guides attention in visual search and influences the speed of detecting angry and happy facial expressions in large arrays of faces. In Study 1, participants were faster to search through clean-shaven crowds and detect bearded targets than to search through bearded crowds and detect clean-shaven targets. In Study 2, targets were angry and happy faces presented in neutral backgrounds. Facial hair of the target faces was also manipulated. An anger superiority effect emerged that was augmented by the presence of facial hair, which was due to the slower detection of happiness on bearded faces. In Study 3, targets were happy and angry faces presented in either bearded or clean-shaven backgrounds. Facial hair of the background faces was also systematically manipulated. A significant anger superiority effect was revealed, although this was not moderated by the target’s facial hair. Rather, the anger superiority effect was larger in clean-shaven than bearded face backgrounds. Together, results suggest that facial hair does influence detection of emotional expressions in visual search, however, rather than facilitating an anger superiority effect as a potential threat detection system, facial hair may reduce detection of happy faces within the face in the crowd paradigm.

## Introduction

People judge emotions from facial expressions^1^, small movements in head position^2^ and facial structure^3^. Facial expressions are phylogenetically ancient and shared across many non-human primate species^4^. In humans, facial expressions are universally performed across disparate societies and while perceptions of some emotions show cross-cultural variation^5^, within cultures, facial expressions are recognisable displays of emotional states^6^.

During human evolution, efficiently processing and correctly responding to threat signals was likely crucial for group cohesion and survival^7^. Indeed, human visual systems rapidly detect signals of environmental threat and reward^8,9^ and faces can serve as such signals^10,11^. For example, the *face in the crowd effect* described how angry expressions are rapidly detected in arrays of faces posing neutral or happy facial expressions due to an evolved pre-attentive threat detection and avoidance system^11^. These findings were replicated in several subsequent studies that renamed the phenomenon—the *anger superiority effect*^8,9,12,13^.

Faces not only convey emotional expressions, but also carry a range of social information (e.g., gender, and ethnicity) that are quickly recognized^14^ and guide subsequent social interactions^15^. First impressions of various personality traits are formed from faces, particularly dominance and trustworthiness^16,17^ that inform important social decisions from voting to criminal sentencing^18,19^. This social information can also influence the way that attention is allocated to faces^20,21^ and can augment the anger superiority effect. For example, the sex of faces influenced the speed of detecting happy and angry faces in crowds^22^. This may reflect that the facial movements required for expressing anger overlap with androgen-dependent craniofacial characteristics, including a robust midface, pronounced brow ridges and thicker jaws characteristic of men^23,24^. As facial masculinity is positively associated with male physical strength^25^, stature^26^, fighting ability^27–30^, and social dominance^31,32^, facial expressions of anger may have evolved to facilitate judgments of male formidability^24^.

Potentially the most visually distinctive secondary sexual trait in males is facial hair^33,34^. Owing to the emergence of facial hair during adolescence and its full expression in adulthood, beards unambiguously communicate age, maturity, and masculinity^32,35,36^. Additionally, using photographs of the same men posing neutral expressions when bearded compared to when clean-shaven, beards increased ratings of social status^37^, social dominance^38,39^, strength^40,41^ and aggressiveness^32,36^. These effects could be due to beards enhancing underlying structural elements of facial masculinity, notably the length of the face and protrusion of the jaw^32,38,42^. In line with predictions that masculine facial shape increases perception of angry facial expressions^23^, facial hair augments explicit ratings of aggressiveness compared to clean-shaven faces^37^ and enhances the speed and accuracy of anger recognition^43,44^. While facial hair may be unrelated to fighting ability^45^, cross-culturally beards are more common among men living under conditions of high male-male competition^46,47^ and potentially in populations with high parasite stress and low health^48,49^. This body of evidence suggests beardedness facilitates anger recognition and threat perception within large social groups.

While many studies have reported anger superiority effects in visual search, some recent studies have reported happiness superiority effects^50^. Prior anger superiority effects may have occurred due to stimulus confounds including poor photographic quality that artificially introduced dark shading and smudges and that when natural stimuli are employed, happiness rather than anger superiority effects emerge^50^. Further, anger superiority effects have been reversed to happy superiority effects simply by using different stimulus sets^51,52^. With regards to the effects of beardedness on recognition of facial emotions, an alternative influence of facial hair is that beards may mask certain facial expressions and reduce the speed and accuracy with which people recognise emotions such as happiness. Indeed, people are slower and less accurate when categorizing facial expressions as ‘happy’ when bearded than clean-shaven^43,44^. Importantly, studies testing anger superiority effects often explicitly controlled for the presence of emotionally irrelevant facial features by removing hair and using clean-shaven faces to remove any potential influence these features could have on results (e.g.,^10,53,54^. Thus, to our knowledge the past 30 years of research into the anger superiority effect in visual search has not tested whether facial hair influences the anger superiority effect.

To this end, the current research examines two related questions. The first aim was to determine how the presence of facial hair influences attention to faces in crowds in the absence of emotional expression. Determining the influence of facial hair on attention, separate from the role of emotional expressions, can aid in the interpretation of studies where both target facial hair and emotion are varied. The second aim was to determine whether the presence of facial hair facilitates the detection of anger in male faces in crowded scenes. Based on findings from past research demonstrating faster allocation of attention to threat signals, it is predicted that (1) participants will be faster to detect bearded than clean-shaven targets in crowds and (2) the presence of a beard will augment the anger superiority effect. Three studies were undertaken. Across all three studies, participants searched for targets within arrays of bearded and clean-shaven faces and responded as to whether a target was either present or absent. To standardise our stimuli and reduce the effects of confounds that have impacted past tests of the face-in-the-crowd paradigm, we adopted a number of Becker et al.’s^10^ methodological recommendations including: (1) Using different identities for each face in the search array to reduce background homogeneity, (2) Using background faces with neutral expressions in studies investigating the anger superiority effect, (3) Matching the composition of backgrounds across different trial types to minimise the impact of low level visual confounds on visual search through backgrounds; (4) Using expressions where teeth were showing for both happy and angry targets to remove teeth presence/absence as a potential confound.

In Study 1, participants viewed homogenous arrays of bearded or clean-shaven faces showing neutral expressions, looking for a target face with differing facial hair (i.e., a bearded face in an array of clean-shaven ones). This initial study aimed to determine whether facial hair modulates attention in crowds of neutral faces. Subsequently, Studies 2 and 3 addressed whether beardedness modulates the anger superiority effect. In Study 2, participants searched through mixed arrays of bearded and clean-shaven faces looking for an emotional target face (either angry or happy and with or without a beard) among neutral faces. In Study 3, participants searched through backgrounds of all bearded or all clean-shaven neutral faces looking for an emotional target face (either angry or happy with or without a beard).

## Study 1

### Method

#### Participants

A total of 52 undergraduate students from Curtin University took part in Study 1 (6 males, 46 females, *M*_Age_ = 22.46, SD = 8.45, range = 17–59). Two participants were excluded due to error rates greater than 25%, leaving 50 participants for the final analysis. This sample size is in line with those in past face in the crowd studies^55^.

### Materials

**Figure 1:**
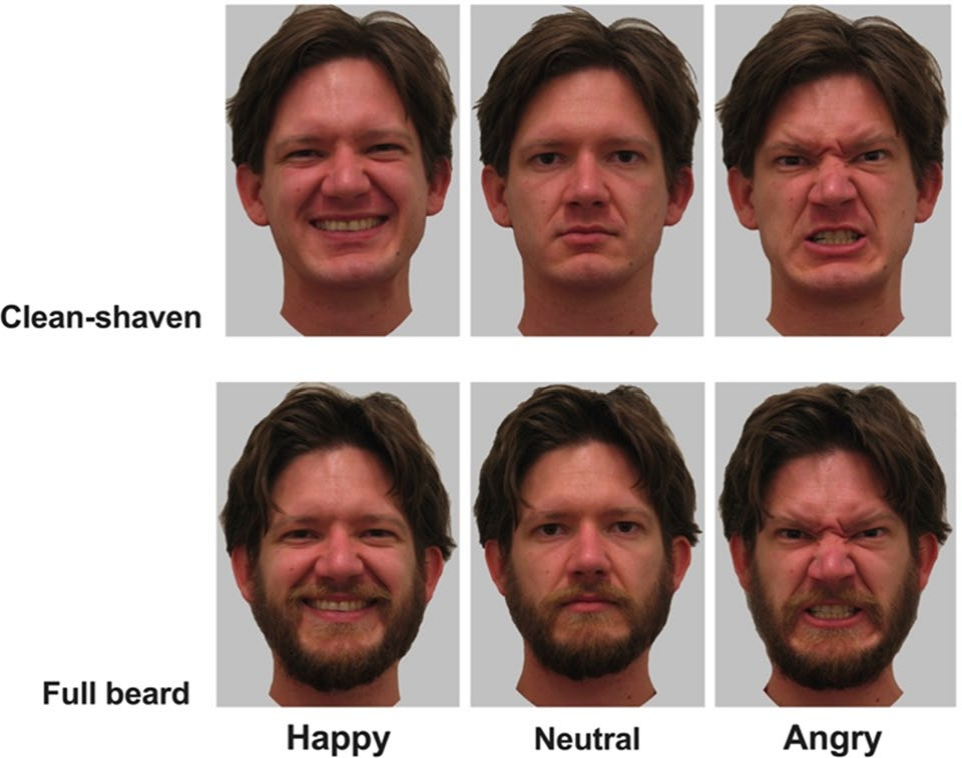
Examples of the stimuli used in Studies 1–3. Clean-shaven faces are shown in the top row and bearded faces on the bottom row. Faces from left to right show happy, neutral, and angry expressions.

Stimuli were photographs of ten young Caucasian males (*M* = 23.50, *SD* = 3.57, range = 20–30) photographed when clean-shaven and bearded posing neutral expressions (Fig. 1). Bearded photographs were taken at 8 weeks (minimum) of untrimmed facial hair. All images had clothing and backgrounds removed and presented on a grey background (Fig. 1). See^37,56,57^ for further details about the creation and validation of this image set and^37,43,44,58,59,60^ for previous ratings of these stimuli.

### Procedure

All experiments were conducted in a computer laboratory. Participants sat at an LCD monitor with a 1920 × 1080 resolution and 60 Hz refresh rate. DMDX software was used to conduct each task and record response times and accuracy^61^. Participants were given verbal instructions by the supervising experimenter and via text-based instructions on their monitor prior to commencing the task. Participants viewed 3 × 3 arrays of faces with each identity represented once and indicated whether all faces were the same regarding their facial hair (i.e., all clean-shaven or all bearded), or if there was a face different from the rest (i.e., a clean-shaven face in a bearded background or bearded face in a clean-shaven background).

Image arrays were presented centred on the screen. A black fixation cross was present in the centre of the screen for 1000 ms followed by the array. Arrays remained on the screen until a response was made or for 5000 ms, after which the next trial commenced. Participants were asked to respond as quickly and accurately as possible, using the left and right shift keys. Response keys were counterbalanced between participants.

**Figure 2:**
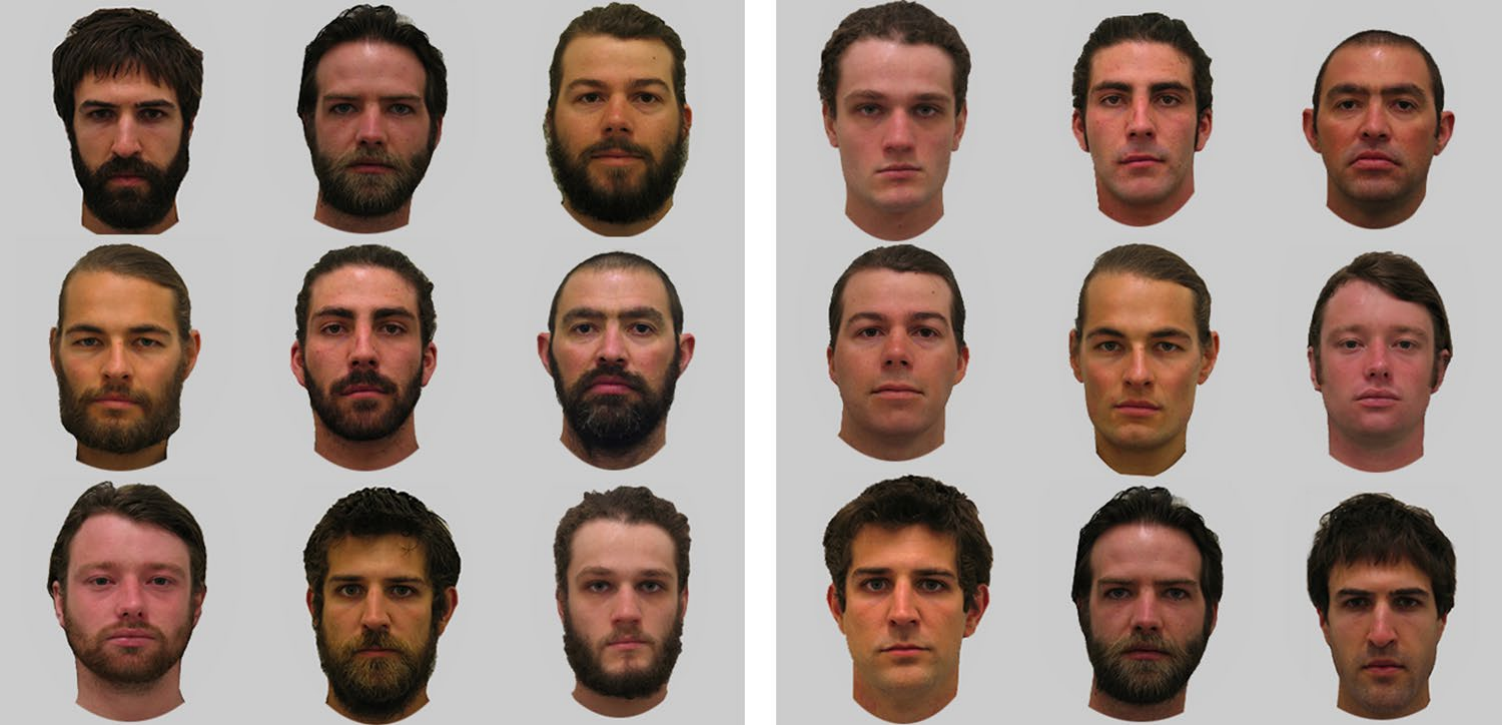
Example search arrays from trials in Study 1. Left: Non-target trial containing uniformly bearded males. Right: A bearded target trial containing one bearded male (bottom row, centre column) in a clean-shaven background.

For all three studies, response times were the dependent variable of interest. All identities and target types appeared in all positions in the array for each experiment. The combination of faces within the arrays repeated across the trial type. Non-target trials were used in each experiment because they require an exhaustive search of the entire array before a target could be declared as absent. Therefore, detection time for target-present arrays should be significantly faster than target-absent arrays. In Study 1, each participant undertook a total of 144 trials (36 non-target trials with all bearded faces, 36 non-target trials with all clean-shaven faces, 36 target trials with a bearded target face in a background of clean-shaven faces, and 36 target trials with a clean-shaven face in a crowd of bearded faces (Fig. 2).

### Analysis

Following previous studies, incorrect responses or response times faster than 100 ms were removed prior to conducting analyses^55,62^ and participants with more than 25% missing responses across all trials were removed^62^. Response times were averaged across each condition and submitted to a 2 (trial: target, non-target) × 2 (target: bearded target, clean-shaven target) repeated measures ANOVA.

### Results

**Figure 3:**
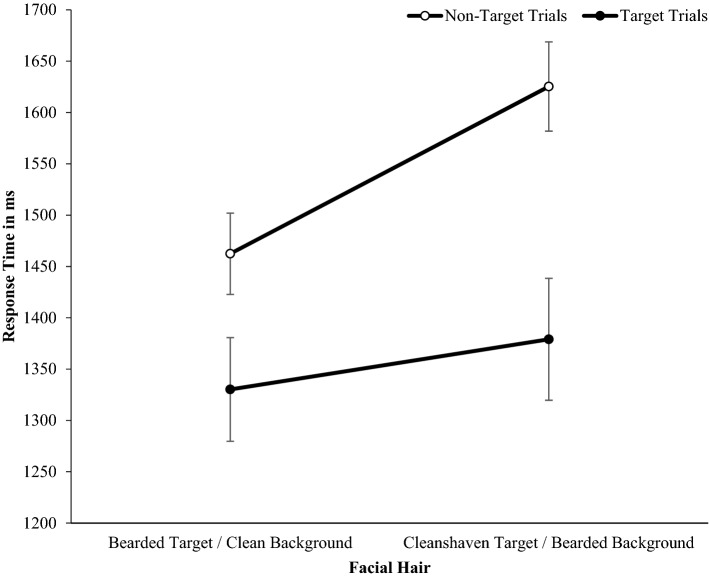
Mean response times for Study 1 (Error bars are ± 1 SEM). The white circles indicate non-target trials, wherein the background does not include a face that is different from the rest (e.g., a clean-shaven background with no bearded faces). The black circles indicate when a face different from the rest in the presence or absence of facial hair was included (i.e., a clean-shaven target in a bearded background).

There was a significant main effect of target type, *F*(1, 49) = 51.20, *p* < 0.001, η_p_^2^ = 0.511, such that participants were faster to respond on target (*M* = 1354.61, *SE* = 40.32) than non-target trials (*M* = 1543.87, *SE* = 53.37). There was also a significant main effect of facial hair, *F*(1, 49) = 30.77, *p* < 0.001, η_p_^2^ = 0.386. Participants were significantly faster to search through clean-shaven arrays for bearded targets (*M* = 1396.29, *SE* = 43.00) than bearded arrays for clean-shaven targets (*M* = 1502.18, *SE* = 49.57). A significant trial type × facial hair interaction, *F*(1, 49) = 14.62. *p* < 0.001, η_p_^2^ = 0.230, revealed participants were significantly faster to detect bearded targets (*M* = 1330.18, *SD* = 279.88) than clean-shaven targets (*M* = 1379.04, *SD* = 307.36), on target trials, *t*(49) = 2.42, *p* = 0.019; *d* = 0.34, and significantly faster to search through clean-shaven backgrounds (*M* = 1462.41, *SD* = 357.01) than bearded backgrounds (*M* = 1625.32, *SD* = 420.04) on non-target trials, *t*(49) = 5.89, *p* < 0.001, *d* = 0.83 (Fig. 3).

### Discussion of Study 1

Participants were significantly faster to respond on target than non-target trials, indicating searches were exhaustive across stimulus arrays to locate target faces. Consistent with predictions, participants were faster to detect bearded target faces than clean-shaven target faces. These findings suggest that a bearded face may be allocated preferential attention in crowds of clean-shaven faces. Results are consistent with the idea that beards may capture and hold attention both as a target and a background distractor. However, on non-target trials, search through clean-shaven backgrounds was also faster than through bearded backgrounds. Given the design of Study 1, we cannot rule out whether faster target detection was due to faster attention to the bearded targets, faster search through the clean-shaven backgrounds or a combination of both^63,64^.

These findings suggest that emotional targets with facial hair may be detected faster in crowds. However, unlike studies investigating the anger superiority effect, Study 1 used only neutral faces and participants searched for targets defined by the presence or absence of facial hair rather than by their emotional expression. Given that the presence of a beard led to relatively faster recognition of anger but slower recognition of other emotions like happiness and sadness in simple emotion categorization tasks (e.g., see^43^, the detection advantage for bearded faces may not be uniform across emotions (or may no longer be present when targets search is guided by emotional expression rather than facial hair). Thus, Study 2 addressed the second hypothesis, which was to determine whether the presence of a beard augmented the anger superiority effect. We adopted a design where participants searched for targets based on emotional expression (the presence or absence of a beard was incidental to participants’ search task). This allowed for the use of identical crowds across different target conditions, so that any difference observed could be attributed to the characteristics of the target rather than the background faces. If beards influence the anger superiority effect, then participants should be significantly faster to detect bearded angry faces over bearded happy faces compared to clean-shaven angry faces over clean-shaven happy faces.

## Study 2

### Method

#### Participants

Forty undergraduate students from Curtin University took part in Study 2 (11 males, 29 females, *M*_*Age*_ = 22.85, *SD* = 6.88, range = 18–47). No participants were excluded.

### Materials

In addition to the neutral stimuli used in Study 1, we also included clean-shaven and bearded faces posing angry and happy expressions, which were posed following the Facial Action Coding System^65^; Fig. 1. See Dixson and Vasey^37^ for further details about the development of this stimulus set.

### Procedure

**Figure 4:**
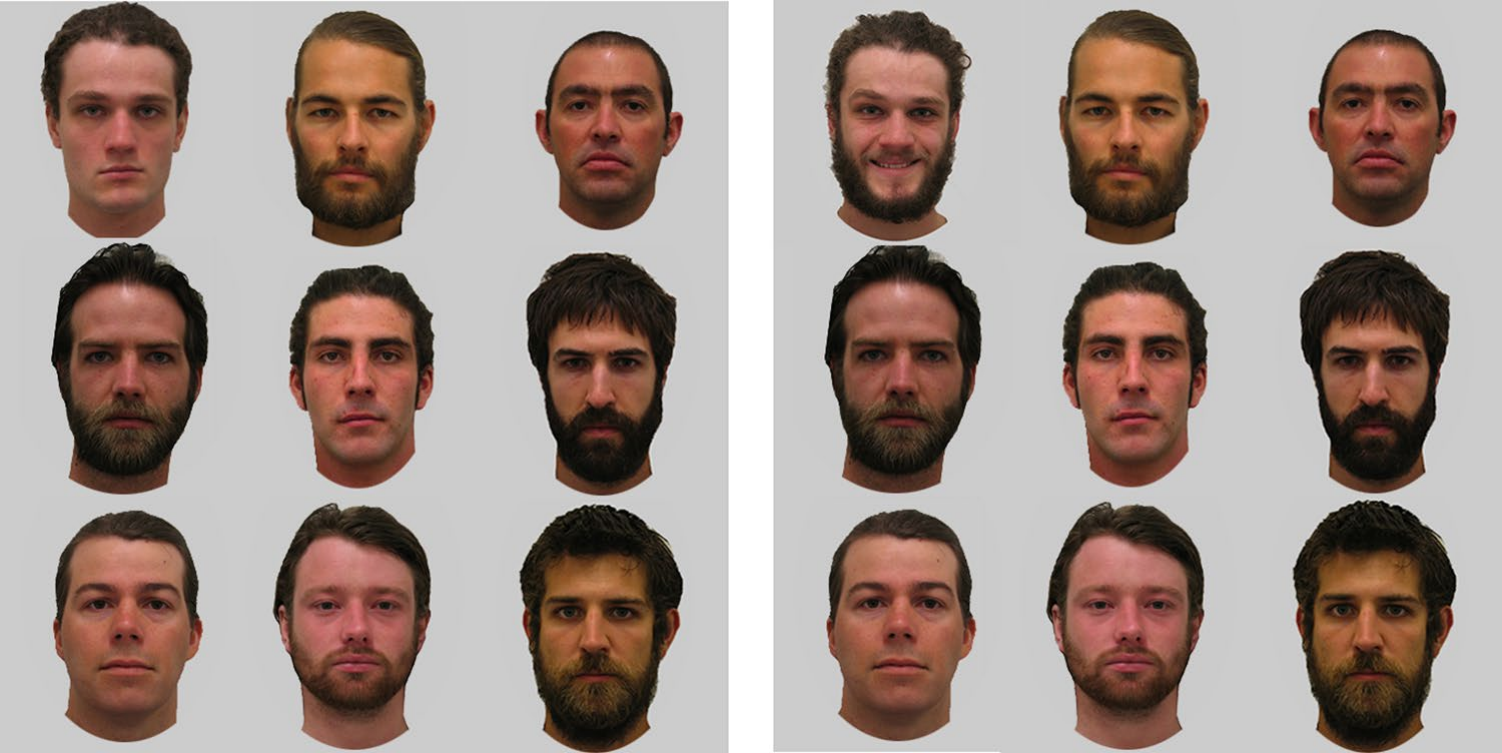
Example of two arrays from trials in Study 2. (Left array) Non-target trial containing uniformly neutral expressions with a mix of bearded and clean-shaven background faces. (Right array) A target trial containing a happy bearded face (top left face) in a mix of neutral bearded and clean-shaven background faces.

The experiment included 180 trials (36 bearded angry target trials, 36 clean-shaven angry target trials, 36 bearded happy target trials, 36 clean-shaven happy target trials, and 36 non-target trials). Participants were asked to determine whether an emotional face was present or absent. In this task, background faces displayed neutral expressions with a mix of bearded and clean-shaven faces represented in the arrays. The number of bearded and clean-shaven faces in the background was randomly determined for each background template meaning that the number of bearded/clean-shaven faces differed between trials. Most trials had roughly the same number of bearded and clean-shaven faces, but some trials had a majority of one facial hair type represented. There were no target trials where the emotional target was also the only face with different facial hair from the rest. Backgrounds were matched across each of the four Facial hair × Emotion conditions so the exact same identities with the same facial hair were presented in the same locations with only the emotional expression and facial hair of the face differing at the target location. This ensured the impact of the visual features of the backgrounds were constant across happy and angry trials. Two versions of the experiment were created (counterbalanced across participants) with different sets of background templates to further reduce any systematic influence of background features on response times. Because of this, any differences observed between conditions could be attributed to the emotional expression and facial hair of the target faces (Fig. 4).

### Results

As in Study 1, response times were averaged across each condition. First, overall response times to target and non-target trials were compared using a repeated-measures t-test. Participants were significantly faster to respond on target (M = 1099.41, SD = 172.84) than non-target trials (M = 1794.37, SD = 352.70), *t*(39) = 19.47, *p* < 0.001, *d* = 3.08. Unlike Studies 1 and 3, non-target trials are not analysed further as non-target trials did not differ systematically by any other factor. All non-target trials were arrays of neutral faces with a mix of bearded and clean-shaven faces present.

**Figure 5:**
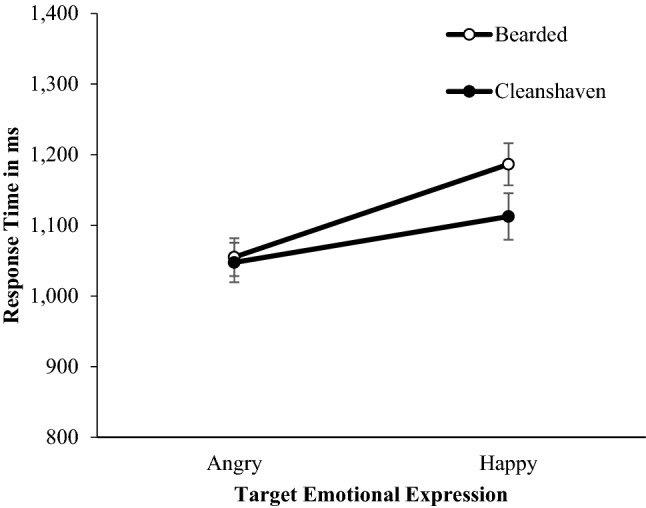
Mean response times for Study 2 indicating the presence or absence of target angry or happy expressions in neutral backgrounds. Error bars are ± 1 SEM.

Second, mean response times from target trials were submitted to a 2 target (bearded, clean-shaven) × 2 emotional expression (happy, angry) repeated-measures ANOVA. For target trials, there was a significant main effect of facial hair, *F*(1, 39) = 12.54. *p* = 0.001, η_p_^2^ = 0.234, whereby participants were significantly faster to detect clean-shaven targets (*M* = 1079.93, *SE* = 29.46) than bearded targets (*M* = 1120.73, *SE* = 26.36). There was a significant main effect of emotion, *F*(1, 39) = 47.71, *p* < 0.001, η_p_^2^ = 0.550, such that participants were faster to detect angry expressions (*M* = 1051.18, *SE* = 26.15) than happy ones (*M* = 1149.49, SE = 30.23). A significant facial hair × emotional expression interaction, *F*(1, 39) = 7.53, *p* = 0.009, η_p_^2^ = 0.162, reflected participants were significantly faster to categorize anger (*M* = 1054.95, *SD* = 169.00) than happiness (*M* = 1186.51, *SD* = 188.51) on bearded faces, *t*(39) = 6.38, *p* < 0.001, *d* = 1.01, and significantly faster to categorize anger (*M* = 1047.41, *SD* = 177.19) than happiness (*M* = 1112.46, *SD* = 208.60) on clean-shaven faces, *t*(39) = 3.93, *p* < 0.001, *d* = 0.62 (Fig. 5), but this anger advantage was significantly larger for bearded targets (Fig. 5).

### Discussion of Study 2

Participants were faster to detect anger than happiness overall, providing support for an anger superiority effect. While participants were also faster to respond to bearded and clean-shaven targets with angry facial expressions than happy facial expressions, this effect was larger for bearded (*d* = 1.01) than clean-shaven (*d* = 0.62) faces, which provides some support for the hypothesis that beards augment the anger superiority effect. However, this facilitation effect appears to be due to slower detection of happy faces when bearded. Although we did not see faster detection of anger on bearded than clean-shaven faces, this finding is consistent with previous research reporting slower recognition of happiness on bearded than clean-shaven faces^43^ and suggests that expressions of happiness are more clearly detected and recognized in the absence of facial hair. Unlike Study 1, participants were faster to detect clean-shaven targets than bearded targets. This is likely a reflection of the change in task demands, with participants searching for a discrepant emotion rather than a face that differed from the rest by facial hair.

In Study 2, all backgrounds were a mix of bearded and clean-shaven faces with neutral expressions. However, only 20% of the trials were non-target trials, so responding was likely biased towards indicating that there was a ‘target present’. Study 3 aimed to test whether slower detection of happiness relative to anger on bearded than clean-shaven faces replicates in a task with a more equal distribution of target and non-target trials, as well as using a design where the facial hair of the faces in the background is also systematically varied. This design also provides an additional trial type to consider. Participants will be presented with trials where there is a face that differs from the rest, but on a task irrelevant dimension. For example, participants will be presented with trials containing a neutral bearded face in a background of neutral clean-shaven faces. These are termed ‘foil trials’ and provide insight into whether participants’ attention is guided by the presence or absence of facial hair even when it does not define the target. If beards influence the anger superiority effect, then participants should be faster to detect angry faces than happy faces overall, with a larger angry advantage observed for bearded than clean-shaven targets. Participants should also be faster to search through clean-shaven backgrounds than bearded backgrounds.

## Study 3

### Participants

356 undergraduate students from The University of Queensland took part in Study 3 (90 males, 241 females, *M*_*age*_ = 19.87, *SD* = 3.73, range = 17–47). Twenty-nine participants were excluded due to errors greater than 25%, leaving 327 participants for the final analysis. The substantially larger sample size occurred as these data were collected along with an unrelated study. Data were accidentally collected for two semesters rather than the intended one semester.

### Materials

The same stimuli used in Studies 1 and 2 were used in Study 3.

### Procedure

**Figure 6:**
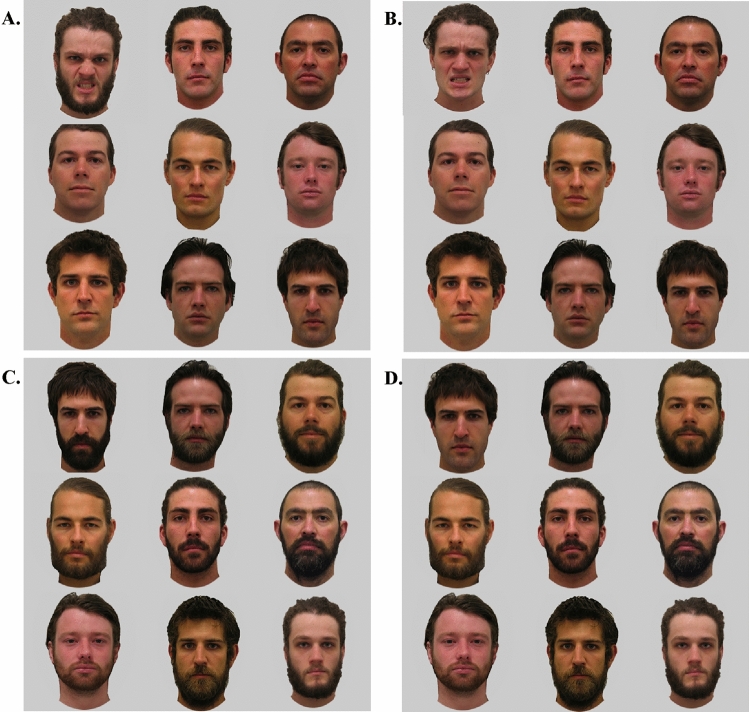
Example of four arrays from trials in Study 3. (**A)** A bearded target angry face (top left face) in a uniformly clean-shaven background of neutral faces. (**B)** Clean-shaven angry target (top left face) in a uniformly clean-shaven background of neutral faces. (**C)** Non-target trial of uniformly bearded neutral faces. (**D)** Non-target trial containing clean-shaven foil (top left face) in uniformly bearded background.

Study 3 had 192 trials: 128 trials had targets present, and 64 trials had no target present. For the target trials, 64 trials had bearded backgrounds and 64 had clean-shaven backgrounds. As in Study 2, participants were asked to determine whether an emotional face was present or absent. Within each of the bearded and clean-shaven background target trials, there was one singleton target face (i.e. bearded or clean-shaven) that differed by its emotional expression (i.e. happy or angry), resulting in 16 trials with each target type (i.e. bearded angry, bearded happy, clean-shaven angry, and clean-shaven happy). On non-target trials, all faces were neutral. On half of the non-target trials, all faces had the same facial hair (16 all bearded, 16 all clean-shaven), and on the other half of the non-target trials there was a singleton (i.e. bearded or clean-shaven) neutral face that differed from the rest (termed a ‘foil’). There were 16 trials where a bearded neutral face appeared in a background of clean-shaven faces and 16 trials with a clean-shaven face in a background of bearded faces (Fig. 6). These trials were presented in order to balance the experimental design, but also to determine whether attention is guided towards faces that are different from the rest on a target irrelevant dimension. For example, participants may be slower to respond on foil trials as the face that differs in the presence or absence of facial hair (the foil) draws their attention. They may initially confuse the foil as a possible target and then have to update their response^66^. This may be pronounced on bearded foil trials given the relatively faster detection of bearded faces in clean-shaven backgrounds in Study 1, as well as the relatively faster detection of bearded than clean-shaven angry faces in Study 2, and the possible conceptual or structural overlap between beards and angry expressions reported in previous literature^43^.

## Analysis and results

### Target vs non-target trials

Response times for target and non-target trials were compared with a within subjects t-test. Participants were significantly faster to respond on target (*M* = 1297.14, *SD* = 309.23) than non-target trials (*M* = 1766.32, *SD* = 464.36), *t*(326) = 23.16, *p* < 0.001, *d* = 1.28. Non-target trials were then submitted to a 2 background (facial hair: bearded, clean) × 2 foil (present, absent) repeated-measures ANOVA. This revealed a significant main effect of background hair, *F*(1, 326) = 18.12, *p* =  < 0.001, η_p_^2^ = 0.053, such that participants were faster to search through clean-shaven backgrounds (*M* = 1747.72, *SE* = 25.85) than bearded backgrounds (*M* = 1784.93, *SE* = 26.25). No other effects were significant indicating that the presence of bearded and clean-shaven foils did not systematically influence search through neutral faces for emotional targets.

### Main analyses

**Figure 7:**
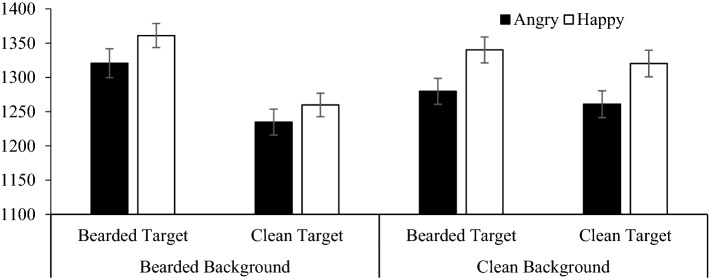
Mean response times (± 1 SE) for Study 3 indicating the presence of target angry or happy expressions, that are bearded or clean-shaven, in backgrounds of uniformly bearded and clean-shaven faces.

We tested the hypothesis that beards slow the detection of happy expressions relative to angry expressions. For these target trials, mean response times were the dependent variable in a two background (facial hair: bearded, clean-shaven) × 2 target facial hair (bearded, clean-shaven) × 2 target emotional expression (happy, angry) repeated-measures ANOVA. The predicted Target emotion × Target facial hair interaction was not significant, *F*(1, 326) = 0.52, *p* = 0.474, η_p_^2^ = 0.002, and there was no three-way background hair, by target facial hair, by emotion interaction, *F*(1, 326) = 0.40, *p* = 0.528, η_p_^2^ = 0.001 (Fig. 7).

**Figure 8:**
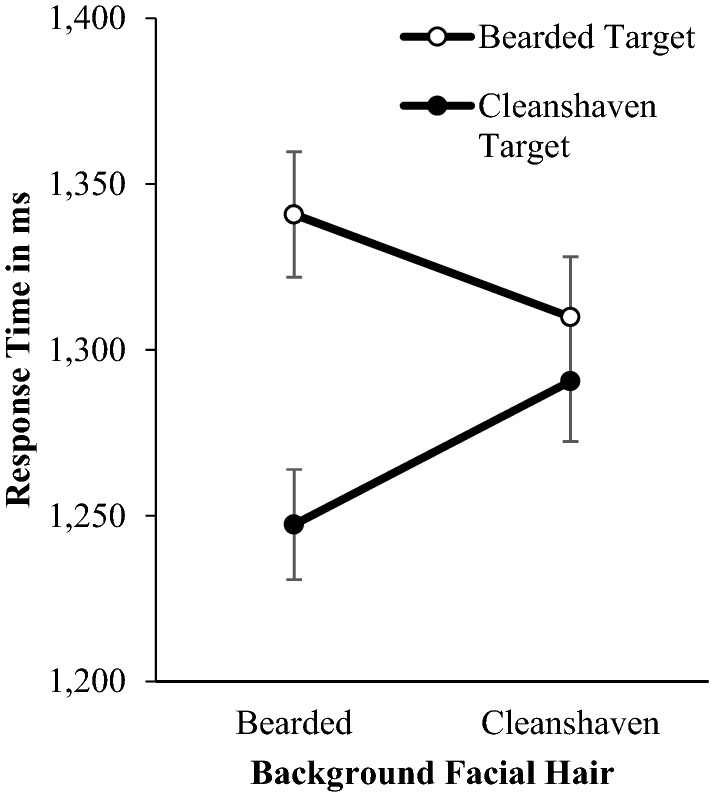
Mean response times in milliseconds (± 1 SEM) for Study 3 indicating the presence or absence of emotional bearded or clean-shaven targets in bearded or clean-shaven backgrounds.

There was a significant a significant main effect of target facial hair, *F*(1, 326) = 90.52, *p* =  < 0.001, η_p_^2^ = 0.217. Participants were significantly faster to detect clean-shaven targets (*M* = 1268.92, *SE* = 16.90), than bearded targets (*M* = 1325.36, *SE* = 17.81), demonstrating that beardedness did not stand out among clean-shaven faces (Fig. 8).

**Figure 9:**
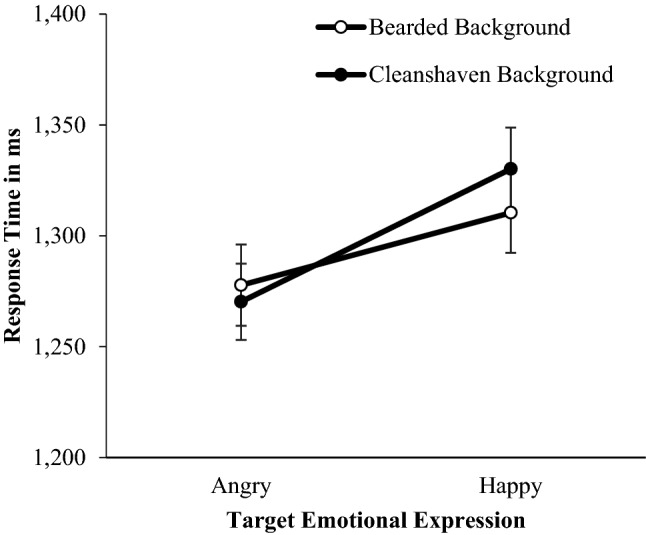
Mean response times in milliseconds (± 1 SEM) for Study 3 identifying target angry or happy expressions which vary in beardedness, in bearded or clean-shaven backgrounds.

A significant main effect of emotion, *F*(1, 326) = 48.47, *p* =  < 0.001, η_p_^2^ = 0.129, revealed that angry target faces (*M* = 1273.99, *SE* = 17.55) were detected significantly faster than happy target faces (*M* = 1320.29, *SE* = 17.30). Response times for targets in clean-shaven (*M* = 1300.20, *SE* = 17.74) and bearded backgrounds (*M* = 1294.07, *SE* = 17.28) did not differ significantly, *F*(1, 326) = 0.66, *p* = 0.417, η_p_^2^ = 0.002. A significant background facial hair × target facial hair interaction was found, *F*(1, 326) = 38.69, *p* < 0.001, η_p_^2^ = 0.106. Participants were significantly faster to identify bearded targets in clean-shaven backgrounds (*M* = 1309.87, *SD* = 329.92), than bearded backgrounds (*M* = 1340.84, *SD* = 342.82), *t*(326) = 2.87, *p* = 0.004, *d* = 0.16. Participants were faster to identify clean-shaven targets in bearded backgrounds, (*M* = 1247.30, *SD* = 300.32), than clean-shaven backgrounds, (*M* = 1290.54, *SD* = 328.18), *t*(326) = 5.22, *p* < 0.001, *d* = 0.23 (Fig. 9).

A significant background hair by target emotion interaction was present, *F*(1, 326) = 5.51, *p* = 0.020, η_p_^2^ = 0.017. A post hoc paired samples *t*-test revealed participants were faster to detect an angry face (*M* = 1277.73, *SD* = 331.34), than a happy face in a bearded background (*M* = 1310.41, *SD* = 310.76), *t*(326) = 3.98, *p* < 0.001, *d* = 0.22. Participants were also faster to detect an angry face (*M* = 1270.25, *SD* = 326.29) than a happy face in a clean-shaven background (*M* = 1330.16, *SD* = 337.29), *t*(326) = 6.38, *p* < 0.001, *d* = 0.35 (Fig. 8), but this anger advantage was larger when targets appeared in a clean-shaven than bearded background.

### Discussion of Study 3

The aim of Study 3 was to investigate whether the larger anger superiority effect observed for bearded faces in Study 2 would be observed in a visual search task with more homogenous backgrounds. Contrary to predictions, although an anger superiority effect did emerge, its magnitude did not differ for bearded or clean-shaven target faces. This discrepancy between the findings of Studies 2 and 3 may be due to differences in study design. For example, the use of heterogenous backgrounds with regards to facial hair in Study 2, but homogenous backgrounds in Study 3 may have contributed to different results. Although the facial hair of the target did not influence the magnitude of the anger superiority effect, there was a significant Background facial hair × Target emotion interaction. This interaction emerged as the anger superiority effect was significantly larger in clean-shaven than bearded backgrounds. This finding suggests that detecting angry expressions is more efficient in clean-shaven backgrounds, perhaps suggesting that angry expressions are more distinctive in the context of clean-shaven than bearded backgrounds.

A secondary aim of Study 3 was to investigate how facial hair may guide attention on non-target trials with and without a foil (a face that differed from the rest with regards to facial hair). Unlike other studies which have found slower response times in the presence of colour or shape foils^66^, this analysis revealed no effect of the presence of facial hair foils. Although analysis of the non-target trials did not suggest an influence of facial hair foils on visual search, the analysis of target trials did suggest that participants used facial hair to guide their search. Following up the significant Background facial hair × Target facial hair interaction indicated that participants were faster to detect emotional targets when they also differed from the background by their facial hair. Together, the results of Study 3 suggest an influence of facial hair on search for emotional targets, though unlike Study 2, the facial hair of the emotional target did not influence target detection speed.

## General discussion

Early empirical studies provided compelling evidence that attention is allocated more efficiently to signals of threat, notably angry faces, than prosocial signals like smiling faces^11^. As facial hair increases perceptions of men’s dominance and masculinity^37,43^ and accentuates the structural features that comprise angry facial expressions^32,38,42^, we conducted three studies investigating whether facial hair moderates the anger superiority effect in visual search.

In Study 1, we aimed to determine whether the presence or absence of facial hair alone alters the allocation of attention to faces. Participants were faster to identify bearded faces in arrays of clean-shaven faces than vice versa. Participants were also significantly faster to search through arrays of clean-shaven faces than arrays of bearded faces to determine target absence. These findings are consistent with cross-cultural studies reporting that the saliency of facial hair as a visually conspicuous cue of masculinity and dominance is stronger under conditions of high male-male competition and in areas of low health^47–49,67^.

Finding faster detection of bearded faces in clean-shaven backgrounds than vice versa, however, can also be reconciled within the broader visual search literature (e.g., Feature Integration Theory^68^). Although faces are complex visual stimuli, in Study 1, the target face always differed from the rest by a single visual feature (the presence or absence of a beard). With the Caucasian stimuli used, beards create a darker patch on the face that contrasts with the models’ fairer skin and thus could be considered comparable to a feature search task (where the target is defined by a single feature [e.g., colour or shape] rather than the conjunction of features). Feature search conditions tend to facilitate efficient search, and in some cases the target will ‘pop out’ from the background. Pop outs occur when target detection is not slowed by an increase in the number of items in the search array as these background items can be processed and identified as non-targets in parallel^68–70^. Finding faster detection of bearded faces in clean-shaven backgrounds than vice versa is consistent with a previously establish search asymmetry where search for the presence of a feature is more efficient than search for its absence^71^. Comparing response times on target and non-target trials suggests that faster detection of bearded faces may be due to faster processing of clean-shaven background faces. This finding, as well as the finding that response times on non-target trials are slower than on target trials, are patterns consistent with a self-terminating serial search strategy. Self-terminating search suggests participants inspect each item in the search array progressively until a target is identified (or not). Although results provide an indication of the use of serial search, all search arrays across studies contained nine faces. This was done to manage the demand on participants in this initial attempt to investigate the influence of facial hair on visual attention, but it also meant that search efficiency could not be calculated to determine whether search relies more on parallel or serial search processes^68,73^). Future research varying array size will be needed to further investigate the contribution of serial or parallel search processes in the detection of targets defined by facial hair.

Taken together, the findings from Study 1 suggest that, when searching for faces that differ from others by the presence/absence of facial hair, clean-shaven faces are processed more efficiently, resulting in faster detection of bearded targets^68–70^. While it remains possible that low level visual features (such as shading or contrast differences) between bearded and clean-shaven faces contributed to differences in response time, rather than the signal conveyed by the beard, this is unavoidable while using naturalistic images of real people with and without beards. Like the literature investigating detection of emotional expressions, future research could trade off ecological validity for stimulus control by using line drawn/schematic faces or digitally manipulated images (e.g.,^63,73^). Indeed, previous studies have used composites of the same men photographed when clean-shaven for studies measuring perceptions of male sociosexual attributes (e.g.^46,74–76^), and could be employed in future replications of Study 1.

In Study 2, we included the same male faces when bearded and clean-shaven. This time targets posed angry and happy facial expressions displayed against backgrounds including a mix of clean-shaven and bearded faces posing neutral facial expressions. The aim was to determine whether the presence or absence of facial hair influenced the presence or magnitude of the anger superiority effect. As predicted, we found participants were significantly faster to detect angry than happy expressions, providing support for past research demonstrating anger superiority effects and rapid perception of threat^11^. The predicted interaction between target facial hair and target emotion was also observed. While the anger superiority effect was larger for bearded angry faces than clean-shaven angry faces, this was due to the slower detection of bearded-happy compared to clean-shaven happy targets. These findings are consistent with a previous study that found participants were slower to detect happiness on bearded than clean-shaven faces^43^. Male faces may more readily communicate anger than female faces due to masculine facial structure overlapping with the muscular movements required to pose anger^24^, while female faces are, on average, more feminine than male faces and enhance perceptions of happy expressions^77^. The incongruency between the masculine craniofacial features emphasised by beards and less masculine craniofacial features on clean-shaven faces that typically underpin happiness could explain the slowed detection of bearded happy expressions. Further, facial hair may mask visual information relevant to recognising happiness, such as the curvature of the lips. A limitation of the experimental design employed in Study 2 was the use of a set of background templates including a mix of bearded and clean-shaven faces. Although this approach meant that any differences observed in response times on target trials could be attributed to the characteristics of the target rather than the background faces alone, it cannot determine how bearded and clean-shaven target detection occurs for emotional expressions when the faces in the background also systematically vary in facial hair.

In Study 3, we combined aspects of the methodologies used in Studies 1 and 2. Like Study 1, participants searched through homogenous backgrounds of all bearded or all clean-shaven faces to detect a target. Like Study 2, participants searched for targets defined by their emotional expression that also differed systematically with regards to facial hair (bearded or clean-shaven). As predicted and consistent with past studies, an overall anger superiority effect was observed^12,13^. Consistent with Study 1, participants were faster to search through arrays of clean-shaven faces to determine target absence. However, contrary to our predictions, and unlike Study 2, no significant interaction between target facial hair and emotional expression emerged. Unlike in Study 2, the anger superiority effect was not significantly larger for bearded targets than clean-shaven targets. The only significant Emotion × Facial hair interaction occurred between target emotional expression and the facial hair of the background faces, reflecting an anger superiority effect in both clean-shaven and bearded backgrounds that was larger in clean-shaven backgrounds. This effect may be consistent with angry expressions appearing more distinctive (or happy expressions less distinctive) in clean-shaven than bearded arrays, in line with the finding that beards enhance anger signals while concealing happy signals^43^.

One explanation for these results is that variation in backgrounds drove differences between tasks. In Study 2, backgrounds were a heterogeneous mix of bearded and clean-shaven faces, whereas in Study 3 the backgrounds were homogeneous with regards to facial hair. Study 3 was not a true conjunction search task as processing the presence or absence of facial hair on background or target faces was not needed to identify the correct response. However, locating the face that differs from the rest by the presence or absence of facial hair would also locate the emotional target or neutral foil on 50% of trials. Detection of these faces may also be facilitated by attention being guided to the face that differs from those surrounding it^78^. This may have led participants in Study 3 to adopt a search strategy that included attending to the presence or absence of facial hair or attempting to attend to the ‘different’ face without consideration of *how* the face was different. Such search strategies would not have been effective in Study 2. Finding that participants were faster to detect bearded targets in clean-shaven backgrounds than bearded backgrounds and clean-shaven targets in bearded backgrounds than clean-shaven backgrounds, is consistent with the idea that participants were attending to facial hair or locating singletons more easily regardless of the facial feature that made the face different from its surrounds. Although additional research would be required to examine this effect, it is possible that heterogeneity in the backgrounds in Study 2 contributed to the interaction between target emotion and facial hair. This may be why Emotion × Facial hair interactions were only observed between target emotion and background facial hair in Study 3.

Together, these studies demonstrate that facial hair plays an important, but nuanced, role in the allocation of attention to faces and that beardedness can facilitate allocation of attention to a target and slow visual search when background faces are bearded. Facial hair also influences the allocation of attention to particular emotional expressions. In Study 2, the effects of facial hair on anger detection over happy detection was stronger than that observed within clean-shaven faces. However, this enhanced anger superiority effect was likely due to the slower detection of happy expressions on bearded faces (Study 2) or in clean-shaven backgrounds (Study 3). Thus, across Studies 2 and 3, the presence of a beard slowed detection of emotional targets, particularly for happiness in Study 2, but for both happiness and anger in Study 3. This suggests that in contexts where many faces are encountered at once, facial hair masks emotional cues making them harder to detect. Previous research has shown slower recognition of sadness and happiness on bearded than clean-shaven faces, potentially due to masking effects of beardedness^43,44^. The current research also suggests that facial hair adds noise to complex visual scenes and can conceal happy emotional expressions more so than angry ones.

There are some important limitations to the current research. For example, attentional bias can occur towards unpleasant over pleasant emotional stimuli^13,79^. Anger is a negatively valanced high arousal emotion that functions to induce submission in others whereas happiness is a positively valanced high arousal but prosocial emotion. The effects of beardedness on detection rates for threatening facial expressions over prosocial expressions may reflect attentional bias towards unpleasant stimuli^13^. In previous research, while the presence of a beard led to faster and more accurate recognition of anger expressions, the presence of a beard slowed recognition of sadness, suggesting beards augment emotional displays of threat rather than stimuli depicting non-threatening unpleasant emotions^43^. Nevertheless, future studies testing whether beards amplify other negative facial expressions, such as sadness, fear, or disgust, in the context of visual search tasks would be valuable.

It is possible that the exposure of teeth could create a significant contrast difference in bearded faces, so that any associations with beardedness increasing detection of angry faces could reflect contrast effects^80^. However, in the current study all happy and angry faces displayed teeth, so the presence of teeth alone cannot explain differences in response times between angry and happy stimuli. A concern in past research attempting to identify anger superiority effects in visual search is that anger superiority effects can be reversed to happy superiority effects by employing different stimulus sets^51,52^. Indeed, Becker and Rheem^50^ recently reviewed the empirical concerns regarding replicability of the anger superiority effect and suggested that when studies employ more ecologically valid facial stimuli, happiness superiority rather than anger superiority effects emerged. Thus, perhaps the most parsimonious conclusion from the present study is that within the context of visual search for emotional faces, beardedness detracts and possibly obscures the signalling potential of happy facial expressions rather than overtly augmenting angry facial displays.

### Ethical statement

Ethical approval for Studies 1 and 2 was granted by the Curtin University Human Research Ethics Committee (reference HR24/2014). Ethical approval for Study 3 was granted by the University of Queensland, Health and Behavioural Sciences, Low and Negligible Risk Ethics Sub Committee (reference 2019000510). All the methods in the current research were carried out in accordance with the relevant guidelines and regulations contained within the institutional ethical guidelines. All photographs were taken by Dr Barnaby Dixson and all participants gave informed consent to have their images used in the current research and reproduced in research led by Dr Dixson.

## Data Availability

Data are available at the Open Science Framework (https://osf.io/5w86u/).
